# ADAM 17 and Epithelial-to-Mesenchymal Transition: The Evolving Story and Its Link to Fibrosis and Cancer

**DOI:** 10.3390/jcm10153373

**Published:** 2021-07-29

**Authors:** Margherita Sisto, Domenico Ribatti, Sabrina Lisi

**Affiliations:** Department of Basic Medical Sciences, Neurosciences and Sensory Organs (SMBNOS), Section of Human Anatomy and Histology, University of Bari “Aldo Moro”, Piazza Giulio Cesare 1, I-70124 Bari, Italy; domenico.ribatti@uniba.it (D.R.); sabrina.lisi@uniba.it (S.L.)

**Keywords:** ADAM17, cancer, autoimmune diseases, inflammation, fibrosis

## Abstract

For decades, metalloproteinase 17 (ADAM17) has been the goal of wide investigation. Since its discovery as the tumour necrosis factor-α convertase, it has been studied as the main drug target, especially in the context of inflammatory conditions and tumour. In fact, evidence is mounting to support a key role of ADAM17 in the induction of the proliferation, migration and progression of tumour cells and the trigger of the pro-fibrotic process during chronic inflammatory conditions; this occurs, probably, through the activation of epithelial-to-mesenchymal transition (EMT). EMT is a central morphologic conversion that occurs in adults during wound healing, tumour progression and organ fibrosis. EMT is characterised by the disassembly of cell–cell contacts, remodelling of the actin cytoskeleton and separation of cells, and generates fibroblast-like cells that express mesenchymal markers and have migratory properties. This transition is characterised by loss of epithelial proteins such as E-cadherin and the acquisition of new mesenchymal markers, including vimentin and a-smooth muscle actin. The present review discusses the current understanding of molecular mechanisms involved in ADAM17-dependent EMT in order to individuate innovative therapeutic strategies using ADAM17-related pathways.

## 1. Introduction

The proteolytic release of transmembrane proteins, the so-called ectodomain shedding, is a crucial step in a wide variety of cellular and biological processes necessary for many fundamental physiological functions, while dysregulated shedding results in detrimental effects on cell behaviour and is linked to severe diseases [1,2]. The Disintegrin and Metalloproteinase 17 (ADAM17) holds in the plasmatic membrane of several cell types and is able to cleave multiple varieties of cell surface proteins [3]. It is somatically expressed in mammalian organisms and represents an indispensable regulator of numerous signalling pathways controlling physiological and pathophysiological processes such as development, regeneration, immunity, chronic inflammation and carcinogenesis [4,5]. Interestingly, ADAM17 might also represent a master-switch during several fibrotic pathologies and has a central role in the regulation of the epithelial-to-mesenchymal transition (EMT), a critical cellular process in cancer metastasis and pathological fibrosis [5]. Numerous studies confirmed that ADAM17 plays a major role in modulating tumour growth and metastasis through regulating cell signalling pathways. In fact, in the progression of carcinomas, epithelial cells lose their characteristics, which are substituted by those of mesenchymal cells through the EMT process, which is induced by the excessive deposition of the extracellular matrix (ECM) and TGF-β signalling [5,6,7,8].

This review provides an overview of the role of ADAM17 in EMT-associated pathways, highlighting new therapeutic perspectives on fibrotic diseases and cancer. As this review underlines, several studies on ADAM17 structure and function in health and disease have been achieved. Being implicated in essential signalling pathways of the immune system, organ fibrosis and cancer progression makes ADAM17 an attractive therapeutic target.

## 2. The Sheddase ADAM17: Biology and Function

### 2.1. ADAM17 Structure

ADAM17 belongs to the family of ADAMs membrane-tethered disintegrin and metalloproteases that cleave cell membrane proteins and/or degrade the extracellular matrix. The human genome contains 20 ADAM genes, and most of the ADAM proteins are proteolytically active [9,10,11]. ADAMs play a key role in the modulation of cell phenotype through their effects on the adhesion, migration and proteolytic activity of many cell types and in the regulating, signalling and responses of cells [10,11].

**Figure 1:**
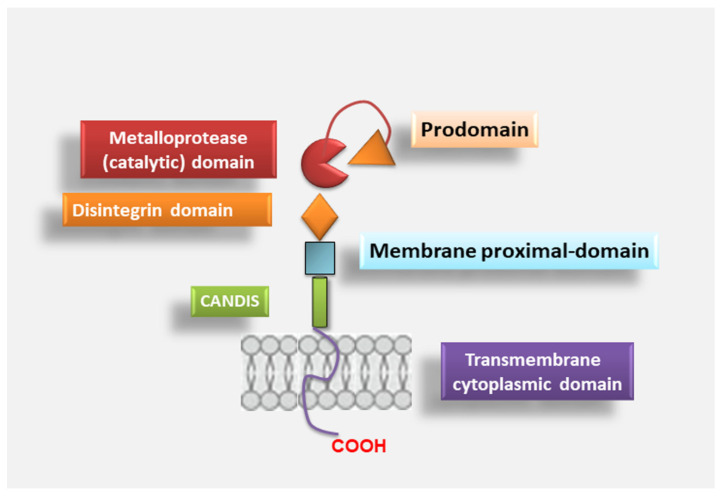
Overview of the ADAM17 structure. The metalloprotease ADAM17 shows six domains that include: *N*-terminal pro-domain followed by a metalloproteinase or catalytic domain, a disintegrin domain, a membrane-proximal domain, a Conserved ADAM-seventeeN Dynamic Interaction Sequence (CANDIS) and ends with a cytosolic tail.

Functional ADAMs mediate the ectodomain shedding of several proteins, such as cytokines, growth factors, receptors, adhesion molecules and endocytic receptors [12]. The ADAMs that are proteolytically inactive are involved in the participation of intercellular communication due to their adhesive properties [13]. Among ADAMs family members, ADAM17, also noted as tumour necrosis factor (TNF)-α converting enzyme (TACE), or better called “molecular scissor”, is the most well-studied protein. ADAM17 was discovered and cloned in 1997 as a metalloproteinase, and Roy Black’s group and others provided direct evidence that cleaves transmembrane TNFα to its soluble form [14,15]. These findings helped to change the significance of ADAMs from simple adhesion molecules to key regulators of cell signalling. ADAM17 is a multi-domain protein composed of 824 amino acids and consists of a series of conserved protein domains that include an *N*-terminal signal sequence (1–17 aa), followed by a pro-domain (18–214 aa) in which there is a cysteine switch-like region, a catalytic domain with a typical HEXXHXXGXXH sequence and a Zn-binding domain region, a disintegrin domain (474–572 aa), a cysteine-rich membrane-proximal domain (MPD) (603–671 aa), followed by a short juxtamembrane segment of 17 amino acid residues that has been named “Conserved ADAM-seventeeN Dynamic Interaction Sequence” (CANDIS), a transmembrane domain (672–694 aa) and a cytoplasmic tail (695–824 aa) [16,17]. The ADAM17 module is depicted in Figure 1.

### 2.2. ADAM17 Activation

**Figure 2:**
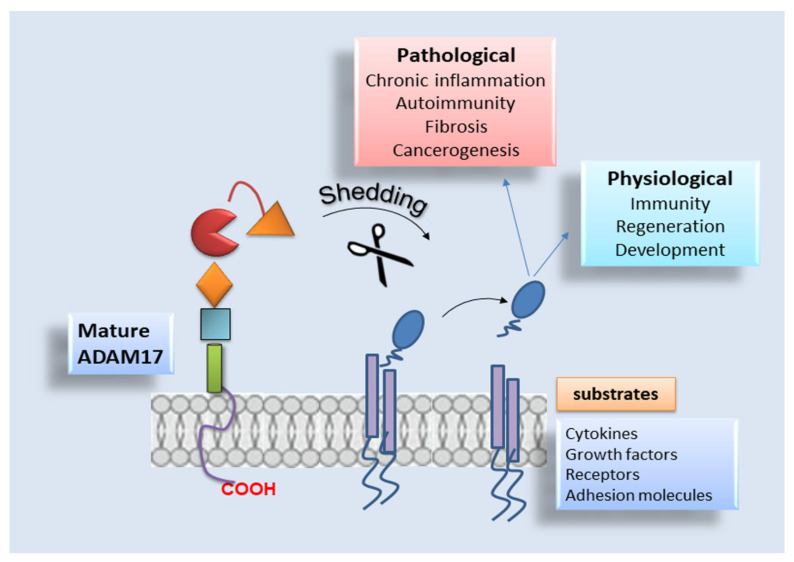
The sheddase ADAM17: schematic representation. ADAM17 sheds 80 several substrates, acting in multiple physiological processes, such as immunity regeneration and development. By contrast, the enzyme acts in various pathological conditions, such as autoimmune diseases, chronic inflammation followed by fibrosis and cancer progression.

ADAM17 is implicated in shedding events controlling the release of several members of the epidermal growth factor (EGF) family, cytokine receptors, critical adhesion molecules and pro-inflammatory mediators [15]. Furthermore, ADAM17 has a central role in many signalling pathways, whereby to perform these activities, it has to be activated and tightly regulated; interestingly, the pro-domain removal resulted in being a prerequisite for its activation [6,18]. Indeed, the pro-domain of ADAM17 preserves it in an inactive form by blocking the metalloproteinase catalytic site [19,20,21,22]. Commonly, the pro-domain of ADAM17 behaves as an inhibitor of the enzyme through the association of a cysteine switch box (SH-group) to the zinc atom in the active catalytic site [23]. The pro-domain is cleaved by furin, a pro-protein convertase, in the trans-Golgi network [23,24] at the last four amino acids (RVKR) preceding the catalytic portion, thus providing the mature form of ADAM17 [25,26]. Studies provided lines of evidence that ADAM17 resulted “packaged” into lipid rafts mediating the transport of the protease to the Golgi apparatus and that this spatial arrangement also led to ADAM17 activity modulation by preserving the protein separated from its substrates [27]. Interestingly, Srour and collaborators, based on in vitro and in vivo studies, demonstrated that the pro-protein convertases PACE-4, PC5/PC6, PC1 and PC2 are able to work as furin-like enzymes that can directly cleave the ADAM17 protein [28]. Finally, after maturation, ADAM17 translocates into the cell surface to perform proteolytic and non-proteolytic functions [29]. Since the pro-domain is highly sensitive to proteolysis, once removed from the catalytic site, it will be destroyed promptly, preventing its re-association with this domain. A schematic representation of ADAM17 activation is reported in Figure 2.

In addition to the pro-domain and catalytic domain, ADAM17 includes other domains for which physiological functions are still widely unknown: a disintegrin domain, which is involved in molecular interactions with other transmembrane proteins such as the integrins; the MPD portion as well as CANDIS can modulate conformational changes and activity by forming electrostatic interactions activity of ADAM17 [30]. Therefore, proximal to this stank region, there is a transmembrane portion involved in ADAM17 interaction with its essential regulators iRhom1 and 2, and an intracellular cytoplasmic domain whose physiological function is still unclear [12].

Recently, research has shown that an early, important regulatory mechanism is represented by the interaction with an adapter factor identified as iRhom1 and iRhom2, pseudoproteases of the rhomboid superfamily, which are essential modulators of ADAM17 maturation and activity [30,31]. Subsequently, it was revealed that iRhom1 plays a central role in ADAM17 maturation, particularly in the brain [32,33]. The iRhom proteins seem to show a role in the selectivity of ADAM17 for some, but not all, substrates [34]. In the last years, various in vitro investigations supplied more accurate knowledge on the molecular relationship between ADAM17 and iRhoms by the use of knockout mice in diverse inflammatory conditions and tumours. Finally, in human patients, mutations in the ADAM17 and iRhom2 genes were identified, which confirmed the importance of the iRhom2-ADAM17 system in immunity and tumorigenesis [32].

### 2.3. ADAM17 Distribution and Substrates

ADAM17 has extensive somatic distribution, being expressed significantly in the brain, heart, kidney, salivary gland and skeletal muscle, and its expression levels vary during embryonic development and adult life [6,14,35,36]. The pivotal importance of ADAM17 in almost every cellular process is established in its different array of substrates represented by growth factors, cytokines, receptors and adhesion molecules. Actually, there are over 80 substrates cleaved by ADAM17, and many of them are involved in chronic inflammation, organ fibrosis and tumour progression. The large repertoire of substrates processed by ADAM17 include molecules that are crucial for tumour immunosurveillance, and the study of the shedding mechanisms coordinated by this protease has led to the proposal of novel events of resistance to noted cancer therapies [37]. As predicted by the notable variety of ADAM17 substrates, gene targeting of Adam17 in vivo led to the death of mice between embryonic day 17.5 and the first day after birth, determining massive developmental defects in the brain, heart, lung, skin, skeletal and immune system [38,39]. Finally, some patients with homozygous mutations in ADAM17 have shown acute and chronic inflammatory diseases such as recurrent severe sepsis, eventually leading to their early death [40]. Given its capability as sheddase, ADAM17 plays a multifunctional role in cancer progression that can vary among different cancer types and phases of the disease. As a consequence of its ability to trigger the EGF receptors (EGFR) pathways by shedding EGFR ligands, ADAM17 activity is linked to several tumours such as colon and breast cancer development [41,42]. Targeting ADAM17, the progression of colon cancer was inhibited in an in vivo model of the disease, determining a reduction in shedding of amphiregulin and the activation of EGFR signalling [43,44,45]. ADAM17 contributes to the development and progression of breast cancer by significant levels of TGF-α, which plays an important role in this pathological process [46,47]. Excess ADAM17 activity contributes to an increased release of EGFR ligands, which can promote cancer evolution, whilst low ADAM17 activity can determine problems in development and regeneration caused by decreased EGFR signalling [48]. Despite all this evidence, however, a complete picture of ADAM17 regulation is still missing (the role of ADAM17 as sheddase is shown in Figure 2).

## 3. The Surprising Role of ADAM17 in the EMT System

Over the last decades, ADAM17 has been reported to be an indispensable key regulator in several biological processes from proliferation to migration. It is, therefore, not surprising that ADAM17, involved in the pathophysiology of numerous human diseases, is critically implicated in EMT [5,6]. EMT is a highly orchestrated process in which epithelial cells shed multiple cellular features linked with epithelial differentiation, including epithelial adherens junctions, apical-basal polarity, cytokeratin expression and the reorganisation of their actin cytoskeleton [49,50]. Furthermore, EMT allows the cells to reach new destinations and generate new cell populations. Concomitantly, the cells undergo a significant dramatic morphological transformation and acquire phenotypes more typical of mesenchymal cells coupled with the enhanced cellular motility characterised by an increased expression of mesenchymal markers, such as E-cadherin and vimentin [51]. EMT can be induced by different extracellular triggers such as various soluble and juxtacrine factors and physical interactions with the ECM through integrin receptors; in addition, the EMT programme can be activated in response to cellular stressors such as hypoxia or therapeutic targets [52,53]. Since ADAM17 mediates the ectodomain shedding of various pro-inflammatory molecules, it is of no surprise that ADAM17 has attracted attention as a potential driver of inflammation and also repurposed pathologically during fibrosis [2,42]. In support of this notion, ADAM17 is overactivated or overexpressed in numerous human chronic inflammatory diseases, and it is noted that EMT represents a convergence point between inflammation and the progression of degenerative fibrotic diseases and cancer [2,42]. Several well-designed studies have shown correlations between the increased levels of ADAM17 expression and the severity of fibrosis in patients with degenerative fibrotic diseases. Furthermore, interestingly, ADAM17 drives several signalling pathways critically involved in the induction of the EMT process [2,42,54]. The specific role of ADAM17 in the pathophysiology of inflammatory and fibrotic diseases is very complex and depends on the cellular context. To exploit the therapeutic potential of ADAM17, it is important to understand how its activity is regulated and how specific organs and cells can be targeted to inactivate or activate this enzyme. For this reason, we undertook this review to reassess the current knowledge on the roles of ADAM17 in the regulation of EMT and, in the following paragraphs, we report the recent insights into potential molecular mechanisms underlying ADAM17-dependent regulation of the EMT process and their relevance to inflammatory, fibrotic and cancer diseases are discussed.

## 4. Mechanism of ADAM17 Signals Modulation in Fibrotic Diseases and Cancer

### 4.1. ADAM17-Mediated Regulation of EMT in Degenerative Retinopathy

The EMT process has been described in proliferative vitreoretinopathy (PVR) and wet age-related macular degeneration (AMD) [55]. PVR is the most common cause of failed retinal detachment repair and is characterised by a sequence of inflammatory and fibrotic mechanisms [56]. Retinal pigment epithelium (RPE) cells, a cellular monolayer composed of mitotically quiescent cells, are known to de-differentiate and lose their fully matured state as a result of a variety of stresses, including oxidative stress and mechanical dissociation of cell–cell junctions [57]. Dissociation of cultured RPE cells leads to morphological changes of the cells into fibroblast-like cells through the activation of the EMT programme [57]. During this process, the RPE cells, trans-differentiated into mesenchymal cells, show increased motility and enhanced ability to proliferate and acquire resistance to apoptosis and the capacity to produce extracellular matrix proteins [57]. RPE cells undergoing EMT contribute to scarring and wound contractions in PVR as well as subretinal fibrosis in advanced AMD [58], which is characterised by the formation of choroidal neovascularisation (CNV) [59]. Several ADAMs seem to be involved in these processes whose expression is variable within the retina; both ADAM10 and ADAM17 are widely expressed during the embryonic period in the different layers of the retina, whereas ADAM12 is mainly expressed in the ganglion cell layer in a later stage of development [60]. The ADAM10, in particular, is activated by TGF-β1 and, specifically, determines the E-cadherin cleavage [61]. The shedding of E-cadherin modulates the activation of the β-catenin signalling pathway, which is involved in the pathogenesis of several fibrotic diseases [62]. Furthermore, both ADAM10 and ADAM17 are involved in the regulation of the Notch-mediated signalling EMT programme important during retina development [63].

Important investigations in this field were conducted by Park et al. on Epstein Barr-transformed adult RPE cells that showed a spindle-like shape phenotype that expresses several mesenchymal markers and secretes TGF-β and VEGF [55]. These cells lose expression of E-cadherin and *N*-cadherin, which is the most common cell–cell junction proteins in RPE cells [64], gaining expression of mesenchymal markers, such as vimentin and/or a-SMA. Using these transformed cells, the authors investigated the molecular mechanisms of EMT in PVR or CNV conditions using the multi-kinase inhibitor Sorafenib (SRF, Nexavar; Bayer HealthCare Pharmaceuticals, Inc., Whippany, NJ, USA) to study the effects on the regulation of EMT by ADAM proteins. SRF, initially approved for the treatment of advanced renal cell carcinoma and hepatocellular carcinoma (HCC), was recently demonstrated to have antifibrotic activity in vitro [65,66,67].

**Figure 3:**
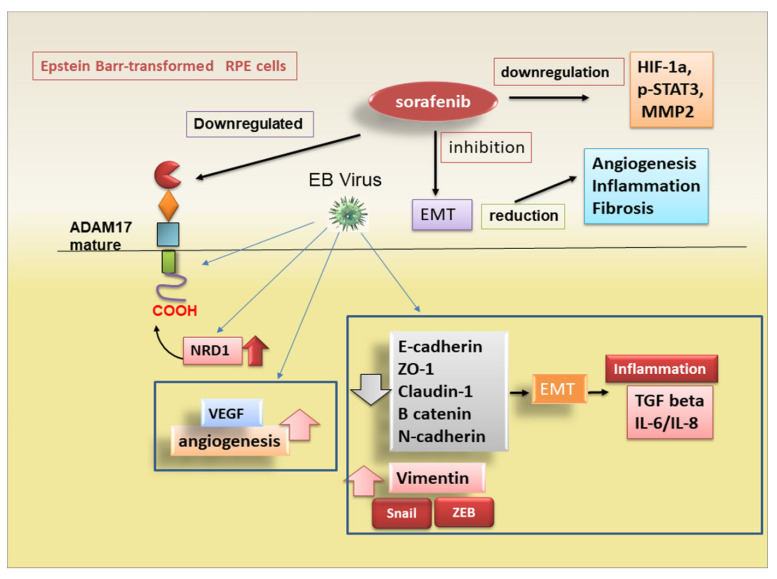
ADAM17 mediates the modulation of EMT in retinal pigment epithelium. Epstein Barr-transformed adult retinal pigment epithelium (RPE) cells express several mesenchymal markers and secrete TGF-β and VEGF. These cells exhibit mesenchymal phenotype features and show a considerable reduction in epithelial markers expression. In these transformed cells was used the multi-kinase inhibitor Sorafenib (SRF) to study the effects on the regulation of EMT by ADAM17. SRF is able to downregulate migration-related signalling molecules, such as HIF-1a, p-STAT3 and MMP2 and, therefore, downregulates the events involved in EMT programme. The expression of mature ADAM17 in RPE/EBV cells was downregulated after treatment with SRF through the regulatory activity of nardilysin (NRD-1).

SRF inhibits STAT3 phosphorylation in a variety of tumours, including HCC [68,69,70]. Moreover, SRF also inhibits TGF-β-induced STAT3 activation during TGF-β-mediated EMT in mouse hepatocytes [67]. Data collected are promising because they show that, in transformed RPE cells, SRF is able to downregulate migration-related signalling molecules, such as HIF-1a, p-STAT3 and MMP2; this process seems to be nardilysin (NRD-1)-dependent. NRD-1, a zinc peptidase of the M16 family localised diffusely in the cytoplasm and secreted to the cell surface [71], binds to the extracellular domain of ADAM17, determining its catalytic activity [72]. This role of NRD-1 was confirmed by NRD-1 knockdown that downregulates the EMT process in EBV-transfected RPE cells [55] (the mechanisms hypothesised are reported in Figure 3). Obviously, the discovery of the precise mechanisms that govern the acquisition of the EMT phenotype from RPE cells in retinal diseases would likely be useful to identify new therapeutic approaches.

### 4.2. Pro-Fibrotic Activity of ADAM17 in Diabetic Nephropaty

Recent experimental evidence reports that ADAM17 is involved in chronic kidney disease (CKD), playing a pro-inflammatory and pro-fibrotic role [73]; blocking ADAM17 activity is, in fact, fibrosis and inflammation resulted attenuated, suggesting ADAM17 as a possible new valuable therapeutic target in CKD treatment. In addition, ADAM17 expression is also variable within the renal parenchyma and seems to be highly expressed in distal renal tubules and increased in the whole kidney in diabetic experimental mice [74].

**Figure 4:**
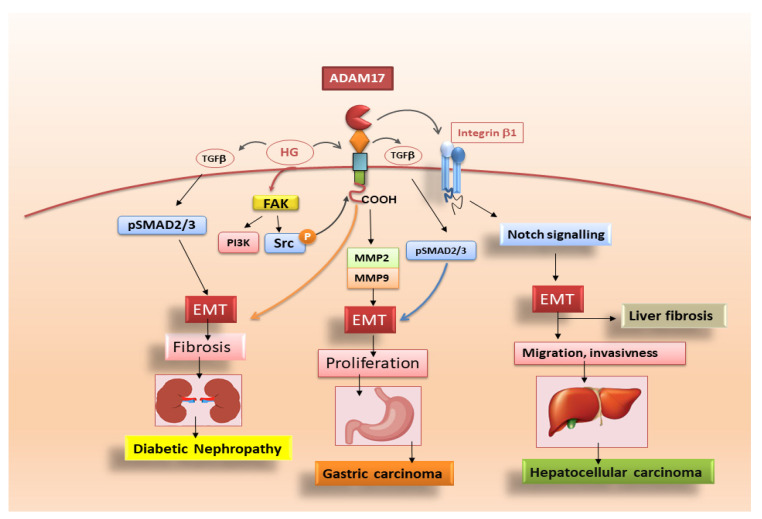
A simplified representation of signalling pathways triggered by ADAM17 in diabetic nephropathy, gastric carcinoma and liver fibrosis. ADAM17 activation is required for high glucose (HG)-induced upregulation of the TGF-β1. HG also induces maturation of ADAM17 on the cell surface. Furthermore, FAK is identified as a central upstream regulator of HG-induced ADAM17 activation through its recruitment of both Src and PI3K, with subsequent phosphorylation of ADAM17. In addition, ADAM17, through an increase in MMP-2 and MMP-9 expression, induces EMT in gastric carcinoma cells. Finally, the role of ADAM17 in promoting EMT of HCC cells involves the activation of Notch signalling pathway, which occurs through Notch proteolytic cleavage.

Diabetic nephropathy is a major cause of chronic kidney disease and kidney failure. Although the kidney undergoes pathological changes in all its compartments, the earliest manifestation of glomerular sclerosis is the deposition of the ECM protein [75]. High glucose (HG) is the base of diabetic nephropathy by determining ECM production in glomerular mesangial cells. TGFβ1 is a major mediator of the HG-induced fibrotic response, and renal cells cultured in HG condition determines phosphorylation and the nuclear translocation of Smad3 [76]. In this scenario, ADAM17 mediates the HG-induced TGF-β1 upregulation and ECM protein production in kidney cells acting on the release of ligands for the EGFR [77,78]. ADAM17 activation is, in fact, required for HG-induced upregulation of the pro-fibrotic cytokine TGF-β1 [78,79]. Researchers demonstrated that the phosphorylation in two C-terminus sites of ADAM17 seems to be involved in the activation of the pro-fibrotic responses of ADAM17. HG also induced furin-dependent maturation of ADAM17 on the cell surface [79]. Furthermore, HG-induced ADAM17 activation requires the upstream regulator focal adhesion kinase (FAK) [79]. FAK acts, recruiting both Src and PI3K, with the subsequent phosphorylation of ADAM17. These studies suggest that the inhibition of ADAM17 activation through targeting HG-specific activators such as FAK or acting on FAK interaction with ADAM17 could represent innovative fields of investigation for the treatment of diabetic nephropathy (Figure 4).

### 4.3. Adam17 Promotes EMT in Gastric Carcinoma

The process of cancer-associated EMT, consisting of the loss of cell–cell junctions, decreases in the epithelial markers, increases expression of mesenchymal markers and cytoskeleton rearrangement, which leads to an increase in cellular invasiveness. Additionally, these changes go hand in hand with the secretion of MMP-2/-9 and FAK [80]. MMP-2/-9 are proteolytic enzymes that degrade the ECM proteins, thus modifying ECM composition and acting directly on cell surface molecules, determining EMT activation [81]. ADAM17 overexpression elevates the expression of MMP-2 and MMP-9, while ADAM17 knockdown downregulates the expression of the same MMPs. This evidence clearly suggests that ADAM17, through the elevation of the MMP-2 and MMP-9 expression, accelerates EMT [7]. ADAM17 knockdown resulted, furthermore, in the downregulation of vimentin, Snail and *N*-cadherin and the upregulation of E-cadherin; in contrast, ADAM17 overexpression led to the upregulation of vimentin, Snail, *N*-cadherin and downregulation of E-cadherin, confirming that ADAM17 promotes EMT in gastric carcinoma cells [7]. As TGF-β/Smad signalling is closely related to EMT in cancer [82], recent investigations reported that ADAM17 knockdown downregulated TGF-β and p-Smad2/3 in gastric carcinoma, while ADAM17 overexpression resulted in the upregulation of TGF-β and p-Smad2/3, but without having any effect on total Smad2/3 protein [7]. Hence, this is a confirmation that ADAM17 promotes EMT probably via TGF-β/Smad signalling in gastric carcinoma (Figure 4).

In conclusion, ADAM17 promotes proliferation, migration and invasion in gastric carcinoma cells. Importantly, the results detail a mechanism (reported in Figure 4) of ADAM17-mediated EMT through upregulating TGF-β/Smad signalling pathway. These findings suggest that ADAM17 might be an important therapeutic target candidate in gastric cancer.

### 4.4. ADAM17-Mediated Mechanisms in Liver Fibrosis

Liver fibrosis is characterised by an excessive accumulation of ECM or scar tissue. The liver resident mesenchymal cells, particularly hepatic stellate cells (HSCs), have been described to be the primary source of ECM in liver fibrosis. As a consequence of liver damage, HSCs start to proliferate and undergo their differentiation into myofibroblasts that have a proliferative, contractile and fibrogenic phenotype [83]. Activated HSCs are characterised by the expression of various specific molecules such as α-SMA, desmin, glial fibrillary acidic protein, platelet-derived growth factor receptor and a massive amount of collagen I [84]. Activated HSCs, once transformed into myofibroblast-like cells, promote chronic inflammation, leading to cirrhosis and HCC. HSCs are major cellular components of HCC stroma, where they modulate the proliferation and invasiveness of cancer cells and are considered the primary source of EMT-dependent fibrogenic myofibroblasts in the injured liver [85,86]. This switch in HSCs differentiation was mediated by ADAM proteases, and different molecular mechanisms have been reported related to ADAM-mediated EMT in the liver [87,88]. ADAM17 performs its role in promoting EMT of HCC through the activation of the Notch signalling pathway, which occurs after Notch proteolytic cleavage and active Notch intracellular domain (NICD) release [89]. Recent discoveries report that the pro-EMT effects of ADAM17 were antagonised by specific micro-RNA whose anti-EMT effects determined a decreased expression of mesenchymal markers (*N*-cadherin and Vimentin) and of pro-EMT transcription factors (ZEB1, SNAIL and TWIST), with a concomitant increased expression of epithelial marker E-cadherin [90]. Based on this evidence, researchers investigated the use of ADAM17 inhibitors, such as ZLDI-8, showing their ability to prevent EMT in HCC cells by decreasing the release of Notch NICD, thereby improving the therapeutic efficacy of anticancer drugs [90,91,92]. Therefore, ADAM17, involved in the transactivation of Notch signalling in liver cancer stem cells, seems to contribute to the enhancement of HSCs aggressiveness [93]. In addition, Notch signalling was implicated in the ADAM17-dependent activation of integrin β1, thereby promoting the proliferation, migration and invasion of HCC cells [94]. In the context of these new findings, a new G-protein-coupled receptor 50 (GPR50)-mediated regulation of ADAM17-induced Notch signalling in HCC progression was also demonstrated [95]. Data collected so far suggest that ADAMs have been implicated at all stages of HCC progression, starting from inflammation and subsequently through to fibrosis, angiogenesis, proliferation, EMT and invasion (Figure 4). The spatial and temporal dynamics of ADAMs activation and their mechanisms of action are, however, still insufficiently characterised, and future work is needed, focusing on better characterising the ADAMs’ contribution to HSCs differentiation in the HCC progression.

### 4.5. Role of ADAM17 in Idiopathic Pulmonary Fibrosis

Idiopathic pulmonary fibrosis (IPF) is a type of interstitial lung disease that is prevalent in elder smokers. The phases of IPF include alveolar epithelial cell damage and activation, inflammatory cell infiltration, EMT initiation and ECM protein accumulation [96]. During the progression of IPF, most fibroblasts originate from lung epithelial cells, which undergo EMT and play a crucial role in fibrotic disease progression. The TGF-β signalling pathway has been suggested to contribute to the EMT process and produce ECM proteins, such as fibronectin (FN) [97]. Therefore, TGF-β and EMT may be a hallmark of fibroblast activation.

As reported above, ADAM17 is responsible for the cleavage of extracellular domains of substrate proteins [54], thus regulating some important physiological and pathophysiological processes and the expression of membrane-bound proteins such as cytokines and growth factors [98]. Increased ADAM17 expression is identified in several inflammatory diseases, cancers, and organ fibrotic changes, including IPF [35,36,99,100]. During the progression of chronic IPF, the volume and ventilation of the lungs are gradually decreased due to abnormal proliferation of fibroblasts through the EMT process, which causes collagen deposition and finally leads to architectural distortion [101]. A recent study demonstrated that ADAM17 regulates TGF-β-mediated EMT through the cleavage of vasorin (VSN), a type I transmembrane protein initially identified in screening to isolate novel proteins containing a signal sequence. VSN was shown to attenuate TGF-β signalling by sequestering the growth factor, and its expression was found restricted to the aorta, kidney and placenta [102,103]. In addition, ADAM17 is responsible for the angiotensin-converting enzyme 2 (ACE-2) ectodomain shedding occurring in lung fibrogenesis, demonstrating that ADAM17 certainly participated in IPF [103]. However, the role of ADAM17 in TGF-β-induced EMT in IPF remains uncertain.

Recently, new impetus has been given to research in this field, finding that connective tissue growth factor (CTGF), an immediate-early protein mediated by TGF-β, regulates the growth of fibroblasts and the secretion of ECM [104]. A previous study suggested that subcutaneous co-injection of TGF-β plus CTGF induced sustained fibrosis in mice [105]. In lung tissue obtained from IPF patients, an enhanced expression of both CTGF protein and mRNA was observed [106], and, moreover, CTGF/integrin-linked kinase signalling mediates the activation of EMT in lung alveolar epithelial cells [107]. Data recently collected revealed an unexpected role for ADAM17 in the regulation of this phenomenon, showing that TGF-β might activate ERK, ADAM17 and Ribosomal S6 kinase-1 (RSK1) signalling pathways; this activation cascade determines the phosphorylation of the enhancer-binding protein β (C/EBPβ) that binds the CTGF promoter region, leading to CTGF synthesis and expression. These investigations start from several lines of evidence of the role of RSK1 and protein kinase C (PKC) in the phosphorylation of C/EBPβ, a transcription factor that participates in the modulation of pro-inflammatory protein expression [108,109]. Moreover, CTGF participates in the mechanism leading to TGF-induced FN expression in human lung epithelial cells [110]; in an experimental model represented by TGF-β-induced renal fibrosis in mice, this mechanism was inhibited, blocking MEK activity and so attenuating CTGF expression [111]; however, it remains unclear as to whether RSK1 and C/EBPβ are involved in TGF-β-induced CTGF expression in human lung epithelial cells and what is ADAM17’s role in EMT activation in the lung. Starting from the results obtained from Blom et al. [104], suggesting that CTGF acts as a modulator of TGF-β-dependent fibrogenesis and EMT activation in lung epithelial cells [104], studies have since progressed; it was demonstrated that TGF-β-induced CTGF expression in human lung epithelial cells provides the participation of ERK, ADAM17, RSK1 and C/EBPβ [110], and both ADAM17 and CTGF seem to mediate TGF-β-induced FN expression [110]. It is clear now the involvement of ADAM17 in the TGF-β-induced expression of CTGF and EMT in the lung; in fact, ADAM17 gene silencing reduced TGF-β-induced CTGF and FN expression in human alveolar basal epithelial cell line [110]. Furthermore, through the use of CTGF gene knockdown, a reduced TGF-β-induced FN expression was observed, confirming the correlated importance of ADAM17 and CTGF in TGF-β-induced FN expression in human lung epithelial cells [110]. Further clarifications on the molecular mechanisms involved in IPF fibrosis emerged through studying the ERK pathway activation that seems to regulate the expression of pro-fibrotic proteins in IPF such as osteopontin [112]. By using a specific ERK inhibitor, TGF-β-induced CTGF expression was reduced in the alveolar basal epithelial cells to levels comparable to those obtained through the use of RSK1 gene silencing; additionally, TGF-β enhanced the phosphorylation of ERK and RSK1 that was decreased by using ERK inhibitors [110]. This experimental strategy allowed for the demonstration that ERK mediates TGF-β-induced ADAM17 phosphorylation, and ADAM17 regulates TGF-β-induced RSK1 phosphorylation; overall, these results suggest that the ERK/ADAM17/RSK1 signalling pathway activation was required for TGF-β enhanced CTGF expression in the lung [110].

The same ERK/ADAM17/RSK1 pathway has a positive effect on C/EBPβ phosphorylation and activation, and ADAM17 plays a key role in TGF-β-induced CTGF expression and EMT through the ERK/RSK1/C/EBPβ pathway [110].

**Figure 5:**
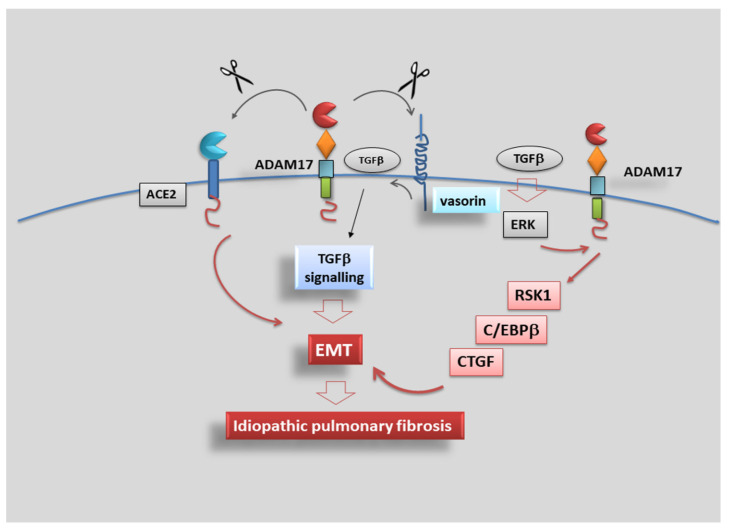
ADAM17 promotes idiopathic pulmonary fibrosis via EMT activation. Representative scheme illustrating the results of TGF-β-induced CTGF expression mediated via the ERK/ADAM17/RSK1/C/EBPβ pathway in human lung epithelial cells. TGF-β activates the ERK/ADAM17/RSK1/C/EBPβ signalling pathway, which finally leads to CTGF expression, promoting EMT in human lung epithelial cells.

In conclusion, data collected evidenced that TGF-β activates the ERK/ADAM17/RSK1/C/EBPβ signalling pathway, after which it promotes the link of C/EBPβ to the C/EBPβ site on the CTGF promoter region to regulate CTGF expression in human lung epithelial cells, revealing a signalling pathway related to ADAM17-dependent EMT and fibrosis, which may provide a new therapeutic orientation for the treatment of IPF (Figure 5).

The ADAM17 substrates of the EMT signalling pathways identified in the above-reported pathologies are summarised in Table 1.

## 5. Future Directions and Conclusions

Since its discovery, ADAM17 has been defined as the “enzyme that does it all”, playing a pivotal role in several areas of cancer and inflammation [54]. However, therapeutic inhibition of ADAM17 has been historically complicated due to its multifunctionality and high similarity with other members of ADAMs and the related MMPs family. ADAM17 is an enzyme ubiquitously expressed in humans, which cleaves more than 80 substrates other than TNF-α. For this reason, its systemic inhibition ends up being organ- or tissue-specific and is regulated by multiple cascade mechanisms; this poses a serious obstacle to the development of a broadly effective antifibrotic or anticancer therapy. To overcome these limitations, several experimental approaches have been utilised to identify molecules able to discriminate between ADAM17 and its relatives and to inhibit ADAM17 in a specific tissue or cell type. Those recently developed derived from the chemical synthesis of highly specific molecules or from the engineering of endogenous inhibitors of ADAM17. In this regard, the discovery of iRhoms showed a revolutionary yet physiological way to selectively inhibit ADAM17, and a novel ADAM17 inhibitor named ZLDI-8 was recently developed through the use of computer-aided drug design and enzyme activity assay, which seems to act by reversing the EMT process through suppressing the Notch signalling pathway.

Based on this experimental evidence, going beyond the natural role in the wound healing response and in the resolution of inflammation, ADAM17 represents a potential source of deregulation in the tumour environment and its therapeutic targeting, when over activated in the tumour environment, either alone or in combination with other immune-modulating therapies, merits investigation. Furthermore, in a promising way, the relationship between ADAM17 activation, inflammation and EMT seems to be an unexpected feature in the progression of organ fibrotic diseases and cancer. Consistent with its role, targeting ADAM17 was shown to have anticancer activity in multiple preclinical systems but, although early phase clinical trials have shown no serious side effects with ADAM17 inhibitors, the consequences of long-term treatment are unknown. Of course, prior to any routine clinical use, the predictive impact of ADAM17 would need to be confirmed in clinical trials. Although it is still complicated and much has yet to be discovered and understood, this should not discourage the effort to identify the gears in the regulatory mechanism of ADAM17 that can guarantee promising therapeutic strategies for the selective modulation of this enzyme.

## Figures and Tables

**Table 1:** Substrates of EMT pathways signalling activation in which ADAM17 has been implicated.

Pathology	Signalling Pathway	Substrates	References
Proliferative vitreoretinopathy	*TGF-β1/EMT signalling*	EGFR ligands	[55]
Diabetic Nephropaty	*TGF-* *β* *1/Smad 2/3/EMT signalling*	Heparin Binding (HB)-EGF	[78]
Gastric carcinoma	*TGF* *β* *1/Smad2/3/EMT signalling*	HB-EGF	[82]
Liver fibrosis	*Notch/EMT signalling*	Integrin B1	[89]
Idiopathic Pulmonary Fibrosis	*ERK/ADAM17/RSK1/C/EBP* *β* */EMT signalling*	Vasorin	[110]
